# Supplementary material for: DNA polymerase α-primase can function as a translesion DNA polymerase
Source: Proc Natl Acad Sci U S A. 2025 Sep 10;122(37):e2517556122. doi: 10.1073/pnas.2517556122 (PMC12452832; doi:10.1073/pnas.2517556122)
Supplement: Supplementary file 1 — Appendix 01 (PDF) [file pnas.2517556122.sapp.pdf]

# Supplementary Appendix

## DNA polymerase $\alpha$-primase can function as a translesion DNA polymerase

By
Ryan Mayle, Roxana Georgescu, and Michael E. O'Donnell

Corresponding author:

Michael E. O'Donnell
Email: odonnel@rockefeller.edu

This PDF Includes:
Table S1
Figures S1-S4

**Table S1 Primer oligos used in this study.**

**Oligo size standards (5’-3’)**

| | |
|---|---|
| **Pr1** | GTCTCGAGCCCATCCTTCCACTTCCCAACCCTCACC |
| **Std1** | GTCTCGAGCCCATCCTTCCACTTCCCAACCCTCACCTTTAAAAAAAAAAAAAAAAAAAAAAAAAAAAAAAAAAAAAAAACACTCTCCAATTCTCACTTCCTACCACATCCCT |
| **Std2** | GTCTCGAGCCCATCCTTCCACTTCCCAACCCTCACCTTTAAAAAAAAAAAAAAAAAAAAAAAAAAAAAAAAAAAAAAAACACTCTCCAATTCTCACTTCCTACCACATCCCTATTCTAACCACATTAATCTACACCTATATATATATCGCGACTGAG |
| **Std3** | GTCTCGAGCCCATCCTTCCACTTCCCAACCCTCACCCACTCTCCAATTCTCACTT |
| **Std4** | GTCTCGAGCCCATCCTTCCACTTCCCAACCCTCACCCACTCTCCAATTCTCACTTTCTACCACATCCCTATTCTAACCACATTA |

**Template Strand oligos (5’-3’)**

| | |
|---|---|
| **LeadNL-long** | CTCAGTCGCGATATATATATAGGTGTAGATTAATGTGGTTAGAATAGGGATGTGGTAGGAAGTGAGAATTGGAGAGTGTTTTTTTTTTTTTTTTTTTTTTTTTTTTTTTTTTTTTTTTAAAGGTGAGGGTTGGGAAGTGGAAGGATGGGCTCGAGACGTTTTTTTTTTTTTTTTTTTTTTTTTTTTTTTTTTTTTTTT |
| **Lead-Tg lesion** | CTCAGTCGCGATATATATATAGGTGTAGATTAATGTGGTTAGAA(**Tg**)AGGGATGTGGTAGGAAGTGAGAATTGGAGAGTGTTTTTTTTTTTTTTTTTTTTTTTTTTTTTTTTTTTTTTTTAAAGGTGAGGGTTGGGAAGTGGAAGGATGGGCTCGAGACGTTTTTTTTTTTTTTTTTTTTTTTTTTTTTTTTTTTTTTTT |
| **Lead-CPD (CPD = T-T dimer)** | CTCAGTCGCGATATATATATAGGTGTAGATTAATGTGGTTAGA(**CPD**)AGGGATGTGGTAGGAAGTGAGAATTGGAGAGTGTTTTTTTTTTTTTTTTTTTTTTTTTTTTTTTTTTTTTTTTAAAGGTGAGGGTTGGGAAGTGGAAGGATGGGCTCGAGACGTTTTTTTTTTTTTTTTTTTTTTTTTTTTTTTTTTTTTTTT |
| **LeadNL-short** | TAATGTGGTTAGAATAGGGATGTGGTAGGAAGTGAGAATTGGAGAGTGGGTGAGGGTTGGGAAGTGGAAGGATGGGCTCGAGACTTT |
| **Lead-Ab (Ab = abasic site)** | TAATGTGGTTAGAATAGGGATGTGGTAG(**Ab**)AAGTGAGAATTGGAGAGTG**GGTGAGGGTTGGGAAGTGGAAGGATGGGCTCGAGACTTT** |
| **Lead-8-oxo-G** | TAATGTGGTTAGAATAGGGATGTGGTAG(**8oxoG**)AAGTGAGAATTGGAGAGTGGGTGAGGGTTGGGAAGTGGAAGGATGGGCTCGAGACTTT |

**Oligos for priming M13mp18 ssDNA**

| | |
|---|---|
| D25 DNA Primer | GTTAAAGGCCGCTTTTGCGGGATCG |
| R25 RNA Primer | rGrUrUrArArArGrGrCrCrGrCrUrUrUrUrGrCrGrGrGrArUrCrG |

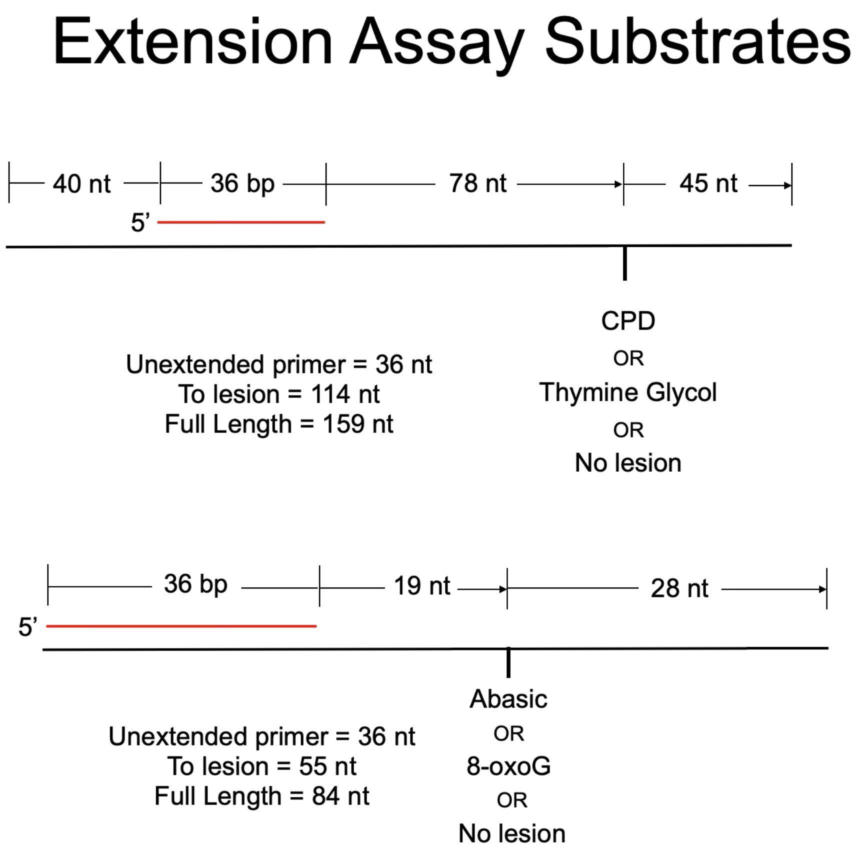


**Figure S1: Diagrams for primer extension substrates**. Details of the sizes for each section of the substrates used in our assay along with lengths of benchmark products. The primer, in red, is 5’ end labeled with $^{32}$P-ATP.

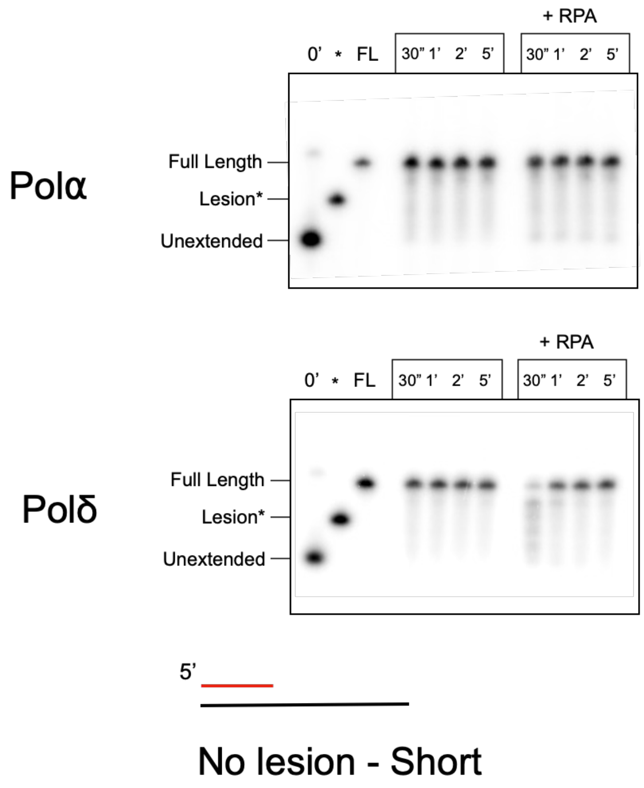


**Figure S2: Primer extension assays on the short no lesion substrate.** Extension products generated by Polα or Polδ with and without RPA. The * indicates products of a size corresponding to extension up to a lesion in the equivalent lesion substrates. This is included as a reference to show no pausing occurs at this location when no lesion is present. The $^{32}$P-ATP end-labeled primer is indicated in red.

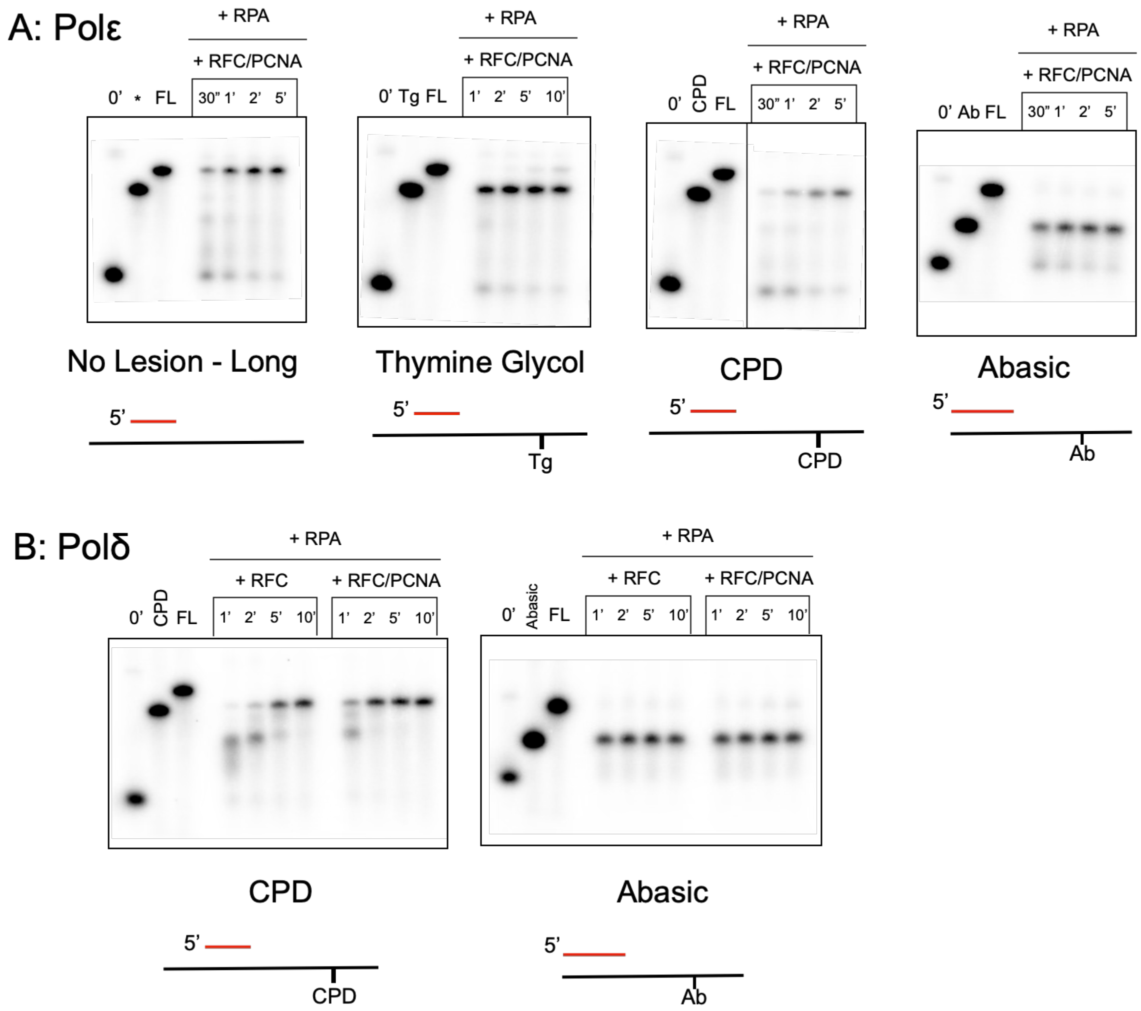


**Figure S3: Impact of RFC +/- PCNA on extension and lesion bypass.** Extension products are shown for assay with Polε or Polδ on the indicated substrate. In all cases, RPA was first loaded followed by initiation of extension by addition of polymerase, RFC, PCNA, ATP, and dNTPs. The * lane in no lesion substrate experiments indicates the size of products where stalling at a lesion would occur in the lesion carrying substrates. Red primers are $^{32}$P-ATP end-labeled.

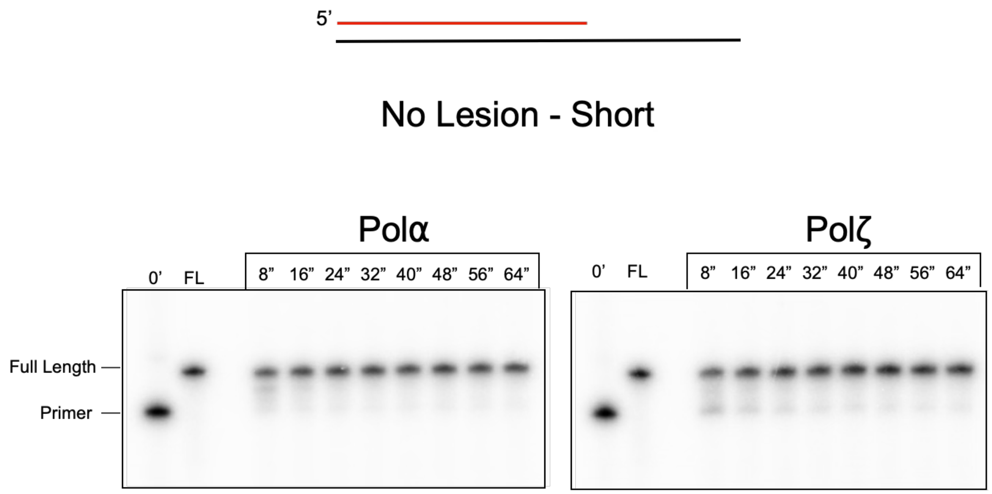


**Figure S4: Primer extension on the short no lesion control substrate.** Extension products from assays with Polα or Polζ at indicated timepoints. Unlike previous experiments, these are primed one nucleotide upstream of the site of lesions in parallel substrates. This illustrates that the 28 nucleotides of extension is completed very quickly. The red primer strand is $^{32}$P-ATP end-labeled.
